# Effect of Processing Parameters on the Mechanical Behavior of 3D-Printed Basalt Moon Dust Reinforced Polylactic Acid Composites

**DOI:** 10.3390/polym17192685

**Published:** 2025-10-04

**Authors:** Lucian Alexander-Roy, Meelad Ranaiefar, Mrityunjay Singh, Michael Halbig

**Affiliations:** 1 Department of Mechanical Engineering, Case Western Reserve University, 10900 Euclid Avenue, Cleveland, OH 44106, USA; lucian.alexander-roy@case.edu; 2 NASA Glenn Research Center, 21000 Brookpark Road, Cleveland, OH 44135, USA; meelad.ranaiefar@nasa.gov; 3 Ohio Aerospace Institute, 22800 Cedar Point Road, Cleveland, OH 44142, USA; mrityunjay.singh-1@nasa.gov

**Keywords:** 3D printing, in situ resource utilization, composites, regolith simulant

## Abstract

Advanced composite materials and manufacturing technologies are critical to sustain human presence in space. Mechanical testing and analysis are needed to elucidate the effect of processing parameters on composites’ material properties. In this study, test specimens are 3D printed via a fused-filament fabrication (FFF) approach from a basalt moon dust-polylactic acid (BMD-PLA) composite filament and from pure PLA filament. Compression and tensile testing were conducted to determine the yield strength, ultimate strength, and Young’s modulus of specimens fabricated under several processing conditions. The maximum compressive yield strength for the BMD-reinforced samples is 27.68 MPa with print parameters of 100% infill, one shell, and 90° print orientation. The maximum compressive yield strength for the PLA samples is 63.05 MPa with print parameters of 100% infill, three shells, and 0° print orientation. The composite samples exhibit an increase in strength when layer lines are aligned with loading axis, whereas the PLA samples decreased in strength. This indicates a fundamental difference in how the composite behaves in comparison to the pure matrix material. In tension, test specimens have unpredictable failure modes and often broke outside the gauge length. A portion of the tension test data is included to help guide future work.

## 1. Introduction

Artemis’ planned return to the lunar surface will require a high level of self-sufficiency to sustain a permanent presence. A promising solution for obtaining the many resources needed for a permanent lunar presence in space is in situ resource utilization (ISRU). A significant portion of the cost for nearly any space mission is in the rocket launch, and even with recent evolutions in commercial spaceflight, the cost per kilogram to orbit is still around USD 1.5–5k [1]. ISRU approaches seek to cut down on launch costs by developing equipment to convert raw materials available on the Moon into water, fuel, and more. Lunar regolith (the fine, ashy material which covers the surface of the moon) has been proposed as a material for applications like construction and radiation shielding [2,3]. Ilmenite, one of the moon’s most common minerals, has been shown to have semiconducting properties [4], which could allow for the in situ manufacturing of solar panels. Several missions have already set out to explore these capabilities. NASA’s Lunar Reconnaissance Orbiter (LRO) has captured extensive scans which indicate the presence of water ice in certain locations just below the lunar surface [5]. The Regolith and Ice Drill for Exploring New Terrain (TRIDENT) carried aboard the PRIME-1 mission took surface samples to analyze gases trapped within the regolith for future use in manufacturing propellant or breathable air [6]. Additionally, additive manufacturing (AM) is being developed as one of the most promising strategies to conduct manufacturing which utilizes the resources available on the moon [7].

The most widespread AM strategies use thermoplastic feedstock in a fused-filament fabrication (FFF) process. FFF produces near-net shape polymer parts with minimal post-processing, using relatively simple hardware, such that it is a capability currently aboard the International Space Station (ISS). The most common materials for this process are polylactic acid (PLA) and acrylonitrile butadiene styrene (ABS). PLA is notable for several reasons: it is derived from corn starch and does not require enzymes to catalyze its reaction, making it easy to manufacture. It is biocompatible, compostable, and can be easily engineered to be amorphous or crystalline depending on application needs [8]. ABS has similar mechanical properties to PLA, and is the primary material used by the ISS’s printer. Reinforcement materials may be used to enhance the properties of FFF parts. This is primarily performed with fiber reinforcement, such as natural fiber or carbon fibers [9,10]. Pre-impregnated filaments can be printed with minimal adjustment to the traditional FFF process. Stronger parts can be produced with more complex hardware, such as the Markforged Onyx platforms, which lay continuous fibers into the matrix during the printing process. Using this approach, Tian et al. was able to achieve flexural strengths as high as 335 MPa in a continuous carbon fiber reinforced—PLA specimen [10]. In addition to fiber reinforcement, the particle reinforcement of PLA has been investigated for impact on tribological, structural, and mechanical performance [11,12,13]. Ranaiefar et al. [13] highlighted the importance of printing orientation and the trade-offs between different filler materials and mechanical strength. Vishal et al. utilized small volumes of silicon filler to achieve compressive strengths of 60 MPa and a tensile strength of 95 MPa [14].

For the AM of non-polymer materials, powder bed fusion processes are the most common method to produce metallic and ceramic parts. Selective laser sintering and selective laser melting have been used in the development of experimental rocket nozzles, and for components on the RS-25 engines for the Space Launch System [15,16,17]. Challenges with the AM of ceramics arise from their very high melting temperatures and difficulty sintering, especially for non-oxide ceramics. One strategy utilizes a binder or matrix with ceramic powder to form a near-net shape ceramic–polymer composite, which can then be pyrolyzed in a furnace to remove the binder and subsequently sintered into a solid ceramic part [18]. The pyrolysis process introduces some shrinkage as the binder burns off, so the dimensional stability of parts must be accounted for during the printing process. However, the uniform heating produces more isotropic material compared with selective laser sintering, which can be prone to columnar grain growth [19]. Another benefit of this process is the flexible manufacturing of the green body part. PLA can be utilized as a binder to form a composite filament that can be printed using traditional FFF printers. Other binders like polyolefin require an additional chemical de-binding step but with the benefit of producing higher-density ceramic parts [20].

Lunar regolith is effectively a mix of various volcanic glasses and ceramics, which raises the question of whether the powder-matrix process can be applied similarly. Various sintering processes have been successfully used to densify lunar regolith simulants [21,22,23,24,25,26]. Works by Song et al. [23] and S. Taylor et al. [24] utilize more traditional hot pressing or spark plasma sintering processes, which eliminate the need for a de-binding process, but can be limited in terms of what geometries can be produced. L. Taylor et al. [25] and Wang et al. [26] utilized direct ink writing and laser powder bed fusion, respectively, to additively manufacture semi-dense regolith specimens, showing the potential for higher complexity parts with regolith. However, these methods utilize highly specialized hardware in comparison with FFF. By combining one of the most widespread 3D printing methods (FFF) with sintering processes, we can achieve the straightforward and scalable AM of regolith, ceramics, and other specialized materials.

To enable the use of these materials, properties such as material strength, wear resistance, and density must be understood. Additional factors are introduced by the 3D printing process; the user’s slicing parameters determine much of the geometry of the toolpath, and these settings can affect the mechanical characteristics of the finished product. For example, a reduction in infill percentage can provide critical material savings at the cost of mechanical strength [27]. Certain printing orientations are necessary to print parts with large overhangs but can cause undesirable shear forces along layer lines. With the introduction of secondary particulate phases, interactions between the matrix, dispersion, and print geometry become even more complex. This report investigates the correlation between print parameter settings and the mechanical behavior of a composite filament made of basalt moon dust simulant and PLA (BMD-PLA). Compression and tensile tests of as-printed test specimens of BMD-PLA and pure PLA were conducted to compare mechanical properties. Visual and statistical analyses were applied to observe the effects of varying print parameters on mechanical properties.

## 2. Materials and Methods

### 2.1. Specimen Preparation

Specimens were manufactured from two materials: a composite basalt moon dust (BMD-PLA) filament from Virtual Foundry (The Virtual Foundry, Stoughton, WI, USA), and a standard PLA produced by Raise3D (Raise3D, Irvine, CA, USA). The composition of the BMD-PLA filament is provided in Table 1. The regolith simulant was supplied to the manufacturer by Astroport Space Technologies (Exploration Architecture Corp., San Antonio, TX, USA) and has a similar composition to the MLS-1 high-Ti basaltic ash simulant that has been established as a faithful recreation of samples collected by Apollo 11 [28,29].

Slicing was performed in ideaMaker 4.3.1 (from Raise3D). Both filaments were a standard 1.75 mm diameter. The specimens were printed on a Raise3D Pro 3 machine (Raise3D, Irvine, CA, USA) at 60 mm/s. By manufacturer recommendation, the BMD-PLA prints were printed at 220 °C with a 0.6 mm steel nozzle, and the PLA was printed at 205 °C with a 0.6 mm brass nozzle. Steel nozzles purchased from Raise3D were used for BMD-PLA as the basalt particulates are abrasive and prone to quickly wearing out brass nozzles, creating issues with over-extrusion. The specimens were evaluated in the as-printed state with no secondary processing steps, except for minimal sanding with #3/0 grit sanding paper on the compression specimens’ contact surfaces with the test rig. This was to ensure the specimen surfaces were flush with the fixture and to minimize any point-of-contact stresses.

**Figure 1:**
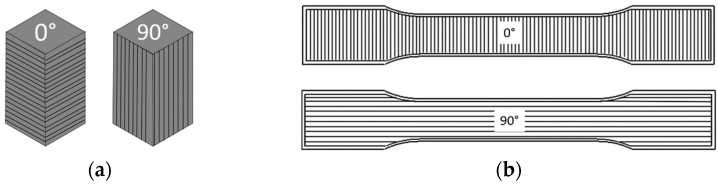
Layer dependence on orientation. (**a**) Compression specimen, (**b**) tensile specimen.

To best explore the effect of multiple print parameters on mechanical properties, a Design of Experiments (DoE) was created for both compression and tensile tests. Many different print parameters are known to influence the desired responses [30,31,32,33,34], including infill percentage, infill geometry, number of walls (shells), layer height, and orientation (load applied parallel or perpendicular to layers). The DoE enables experimental variation in multiple parameters while keeping a manageable number of required tests and is a strong tool utilized in other works where AM parameters are considered [30]. For the compression strength DoE, three parameters were selected at two levels: shells (1|3), infill percentage (50|100), and orientation of layer lines (0|90°). This angle was set relative to the direction of printing, so a 0° orientation has layer lines perpendicular to the direction of compression, and the 90° is in plane with the compression (see Figure 1a). Using a full factorial array, this resulted in eight batches of experimental variation where the sample order was randomized for testing. Batch numbers and their corresponding print parameters are given in Table 2. Although layer height can impact mechanical strength, specifically due to anisotropy, this parameter was fixed at a value of 0.2 mm. Infill geometry can be similarly impactful with variations ranging from simple grids to complex gyroid patterns. However, due to the challenges in quantifying this using a standard DoE, a constant honeycomb structure was utilized in this work. The impact of the infill pattern has been explored by other works, such as those from Birosz et al. [27] and Dudescu et al. [33]. By limiting the number of parameters assessed in this study, the experimental burden of a full factorial DoE was substantially decreased, while preserving statistical power and enhancing the reliability of the findings.

For the tensile DoE, we selected 3 parameters with 2 levels and 1 midpoint: number of shells (1|3|5), infill orientation (0|45|90°), and print layer height (0.1|0.2|0.3 mm). All samples were printed with solid fill, as typical dog bone tensile specimens are too thin to have significant infill volume [31]. The orientation refers to the direction of the rectilinear pattern in the solid fill; the shell walls retain the same geometry regardless of orientation. The sample order was randomized for testing. Batch numbers and their corresponding print parameters are given in Table 3.

**Figure 2:**
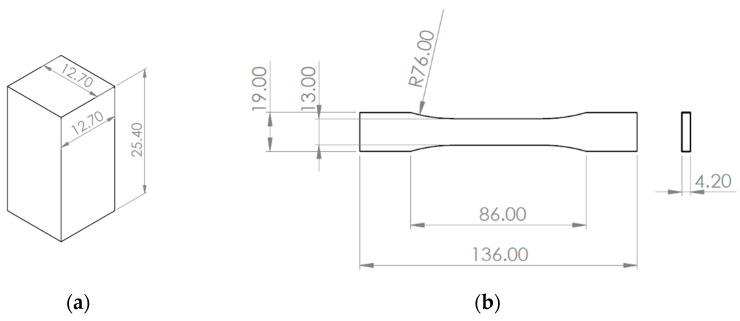
Test specimen dimensions, in mm. (**a**) Compression test specimen dimensions; (**b**) tensile test specimen dimensions.

In accordance with ASTM D695 [35], a rectangular prism compression specimen design was prepared in SolidWorks 2024 (Dassault Systèmes, Vélizy-Villacoublay, France) with dimensions of 12.7 mm × 12.7 mm × 25.4 mm (see Figure 2a) and exported as an STL file. A rectangular specimen was selected over the more common cylindrical geometry, as it allowed for easier adjustment of the print orientation without altering the overall geometry of the specimen. An additional specimen from each batch was prepared for digital image correlation (DIC) to map the strain through localized displacements on the surface of the sample. For these specimens, a coat of white paint followed by a speckle of black paint was applied across the gauge section. In total, 8 batches with 5 specimens each were printed for testing, for a total of 40 specimens per material.

The tensile specimens were prepared with consideration of ASTM standard D638 in SolidWorks with the dimensions shown in Figure 2b [36]. The thickness was chosen to be 4.2 mm as this value allowed us to print at 0.1, 0.2, and 0.3 mm layer heights while maintaining a nominal specimen thickness across all tests. 9 batches with 3 specimens each were printed for testing, for a total of 27 specimens per material.

Pre- and post-compression samples were cut, mounted, and polished for microscopy. Cuts were made parallel and perpendicular to the layer lines on a Techcut 5 (Allied High-Tech Products, Compton, CA, USA), then mounted in two-part epoxy (Metlab Corporation, Niagara Falls, NY, USA). Care was taken to ensure partial infill specimens had the air evacuated from their volume prior to mounting. The mounted samples were first ground down ~3 mm to remove imperfections from cutting, then polished in stages on a Struers Abrapol-30 Polisher (Copenhagen, Denmark) with diamond suspension, in order from 9 µm, 6 µm, and 3 µm to 1 µm.

### 2.2. Testing

Compression testing was performed on an Instron (Norwood, MA, USA) 8562 test stand. Before each test, the mass, length, width, and height of the samples were collected. The tests conformed to ASTM D695 [35], with a compression rate of 1.3 mm/min and a position limit of 13 mm. Load cell position and load data was collected for each test at a polling rate of 10 Hz, and the peak load was manually read off the machine. Most tests were concluded prior to reaching the position limit, as the samples would become too deformed to produce meaningful data.

Tensile testing was performed on an Instron 5966 test stand. Specimens were similarly massed and had area measurements taken around their gauge lengths. A mechanical extensometer from MTS (Eden Prairie, MN, USA) was used to measure strain. The tests were run in accordance with ASTM D638 [36].

### 2.3. Visual Analysis

Additional test runs were performed with DIC using an ARAMIS (Trilion Quality Systems, Plymouth Meeting, PA, USA) system. A virtual extensometer plotted load vs. strain alongside a video of the strain map. The imaging rate was 2 Hz for the first 100 stages, then switched to 0.25 Hz. As the compression proceeded beyond the ultimate strength, many of the samples became too deformed for the virtual extensometer to track the surface, and so the load vs. strain plot produced is not complete for some of the tests. However, this is supplemented with the load cell position and load data recorded from the test frame system.

Microscopy was performed using a Phenom ProX desktop SEM (Thermo-Fisher, Waltham, MA, USA) and a Keyence VHX-7000 optical microscope (Itasca, IL, USA). Pre- and post-test samples were imaged to identify variances in microstructure due to loading. SEM images were primarily taken with the settings of 5 kV intensity, image mode, and back scattered detection (BSD Full) detector type.

ImageJ (Version 1.54p 17 February 2025) image processing was used to measure the area fraction of particulates from the SEM images, and to measure the internal cross-sectional area of the 50% infill samples.

### 2.4. Statistical Analysis

**Figure 3:**
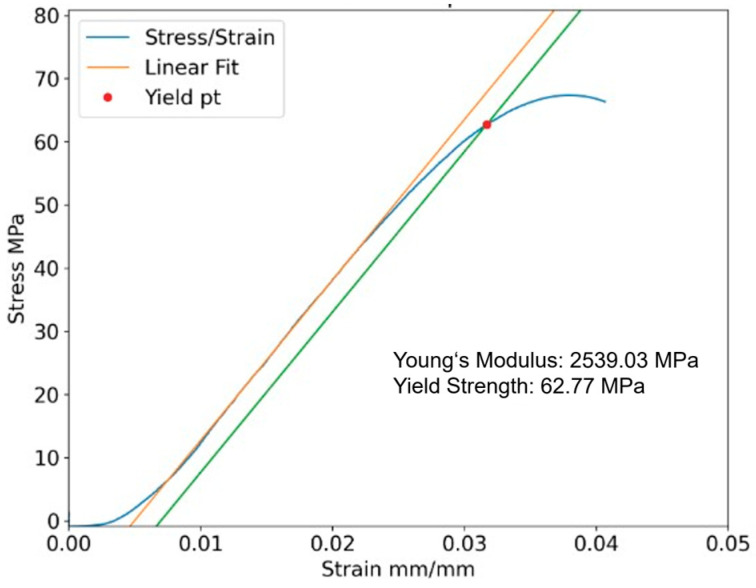
Example compression stress–strain curve of one sample with 100% infill, three shells, 0° orientation.

From the load, position, and dimensional data taken, stress–strain graphs were plotted. Stress is calculated by Equation (1), and strain is calculated by Equation (2). Individual plots were made, which include a calculation of yield strength and Young’s modulus based on the slope of the elastic region. A standard 0.002 strain offset line was used to calculate the yield point (see Figure 3). Excel was used to calculate the mean and standard deviation of yield, ultimate strength, and elastic modulus for each batch in PLA and BMD-PLA, shown in Table 4 and Table 5, respectively. Ultimate strength values for PLA are not reported as the loading would continue to increase even as the plastic began to squeeze outside of the compression surfaces.

(1) σ=F/A

(2) $$\varepsilon =∆L/L_{0}$$

where *σ* is the engineering stress, *F* is the applied force, *A* is the cross-sectional area, *ε* is the engineering strain, Δ*L* is the change in length of the specimen, and *L*_0_ is the gauge length of the specimen.

A 1.5-sigma rejection range is used to check for outliers in the data [37]. A single outlier point was noted in BMD-PLA batch 7, sample 35, which was excluded. A plot of the fitted models vs. the actual data (given in Appendix B.3) confirmed that all other data points were acceptable. Yield strength, ultimate strength, and elastic modulus responses were analyzed via linear regression in Design-Expert software (Version 13.0.12.0, StatEase, Minneapolis, MN, USA) to create Pareto charts of response strength discussed below and in Appendix B. ANOVA was conducted to assess the significance of the models generated. The *p*-values for the three factors and their interactions for both materials are given in Appendix B.1. Models which were automatically adjusted to exclude certain factors or interactions may only have some *p*-values listed. All models and each response listed were within the significance criteria, except for the model for elastic modulus of PLA. The PLA models were not able to account for the variance in the responses (with R-squared values less than 0.5), suggesting that a non-linear regression or addition of other parameters may be necessary to increase the predictive strength.

## 3. Results and Discussion

As seen in Table 4 and Table 5, the pure PLA is significantly stronger than BMD-PLA in compression and tension. For 100% infill compression specimens, the Young’s moduli are similar but slightly reduced from the PLA to BMD-PLA and are near to the accepted values for pure PLA [38]. Collated plots with PLA and BMD-PLA stress–strain curves for each batch are included in Appendix A. Ultimate strength values for PLA are not included as the load would rise continuously up to the position limit set by the test standard. The strengths of each factor (Infill %, Orientation, and Shells) on the response (yield strength, ultimate strength, and elastic modulus) are discussed below and in Appendix A.

**Figure 4:**
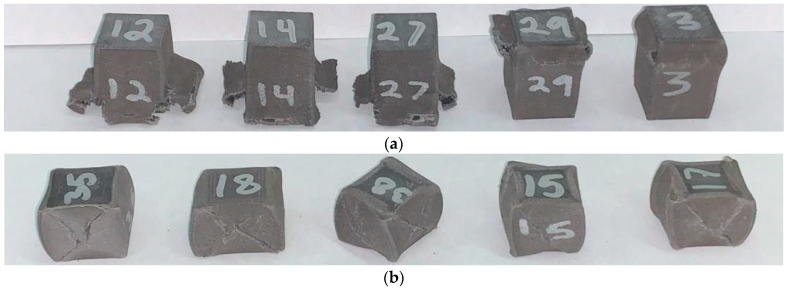
Post-test compression samples. (**a**) Compression batch 1; (**b**) compression batch 7.

Samples displayed a variety of failure mechanisms depending on print parameters used. Figure 4 shows the difference in deformation between 50% infill, shell, with 90° orientation (Figure 4a), and 100% infill, three shells, with 0° orientation (Figure 4b). The 90° orientation samples tended to have their walls peel away from the bulk of the specimen. The 100% infill samples tended to fracture diagonally from the corners.

**Figure 5:**
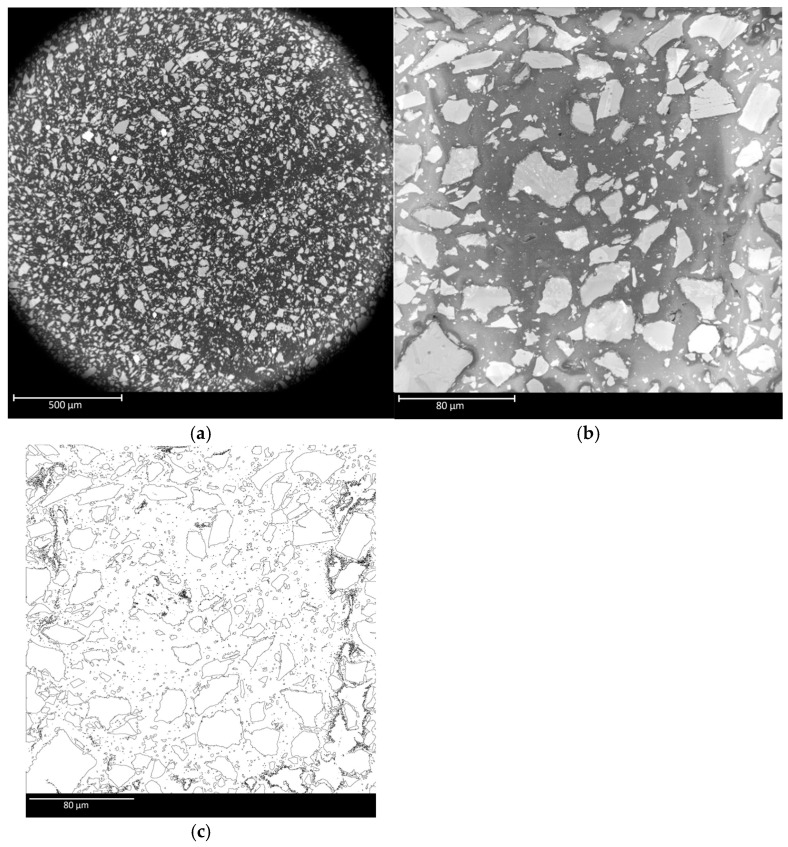
SEM microscopy of printed BMD-PLA sample. (**a**) 150× magnification; (**b**) 1000× magnification; (**c**) particle area fraction trace of 5b from ImageJ.

Microscopy provided some insight into the distribution of the particulates within the matrix (Figure 5). In the pre-compression photos, lighter, jagged regions of the basalt surrounded by the darker PLA matrix are observed. The particle size is in the range of ~2 µm to 40 µm. ImageJ analysis of six SEM images was used to calculate the area fraction of particulates. Additionally, cross-sectional area measurements of the 50% infill samples were taken and used to adjust the stress values for compression batches 1–4. Since the 50% infill samples have internal voids, the outermost dimensions are not reflective of the true cross-sectional area. The results of the particulate area fraction measurements are given in Table 6. The results of the surface area measurements are given in Table 7.

**Figure A6:**
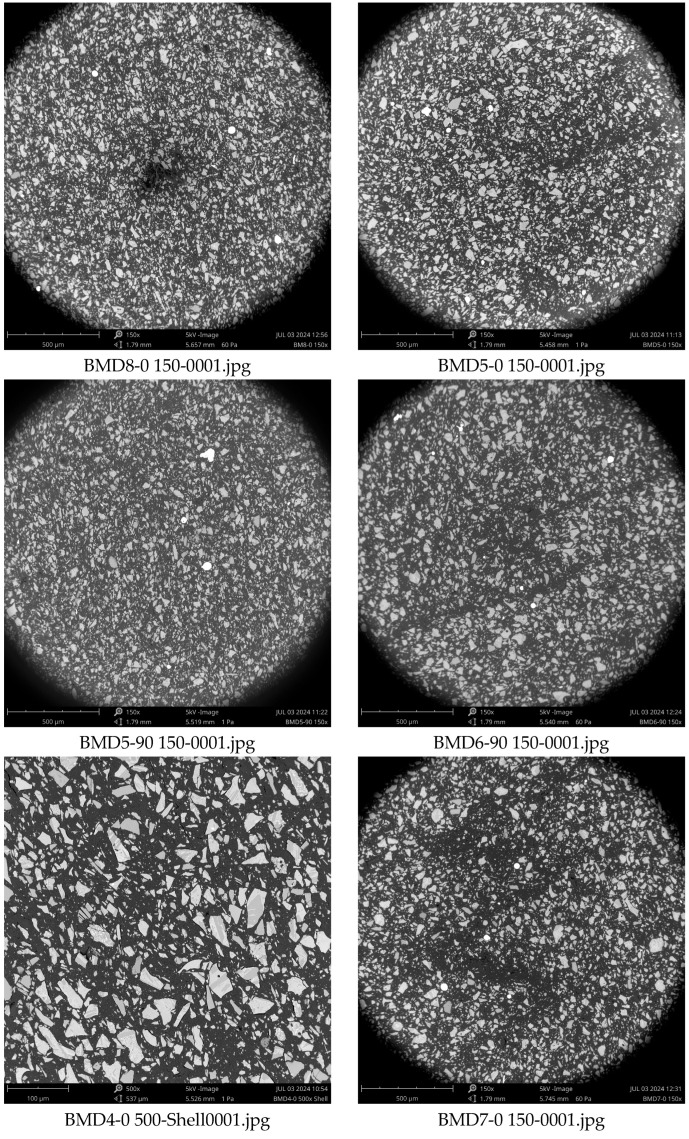
SEM images of various samples used for area fraction analysis.

Typical area fraction measurements in isotropic regions of the material had consistent values around 33%, but microscopy images of border regions between printed lines had reduced area fraction in the 25% range. Images used in the area fraction calculation are included in Appendix C, Figure A6. The adjusted surface area increased the stress and modulus measurements significantly, especially in batch 2.

**Figure 6:**
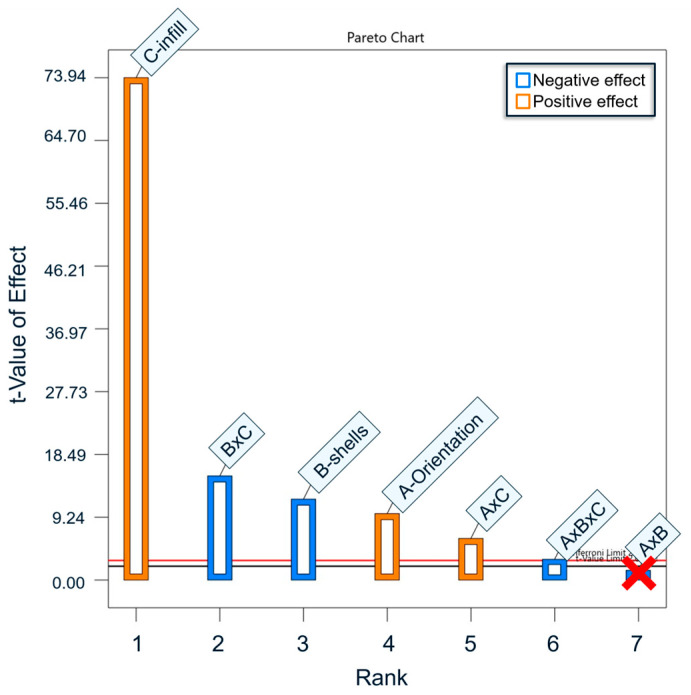
Design of Experiments results: compressive yield strength main effects. A = Orientation, B = Shells, C = Infill. The AxB interaction is crossed out as it was below the significance threshold.

The Pareto Chart in Figure 6 shows the weighted effects of each print parameter and their interactions on the yield strength of BMD-PLA. The t-values represent the magnitude to which each print parameter affected the mechanical response (e.g., yield strength, ultimate strength, and elastic modulus). A high t-value for infill % indicates that print parameter was the largest contributor to the compressive yield strength of the specimens. Infill % was the strongest factor in determining compressive yield strength in the BMD-PLA samples. Aligning the print layers with the loading axis had a small contribution to strength. Increasing the number of shells appears to have a detrimental effect, but this is likely a product of the Infill-Shell interaction. Since increased infill has the largest effect on strength, replacing that infill with extra shells results in decreased strength. By contrast, the strongest factor for PLA yield strength was a negative interaction between orientation and infill. The elastic modulus of BMD-PLA was overwhelmingly dependent on infill % but was consistently more rigid than PLA for the same print settings. Ultimate strength exhibited similar dependence with significant contribution from print orientation.

In the compression data, the composite material’s strength is not linearly related to the volume of matrix material. The orientation main effect was positive for BMD-PLA and negative for PLA, meaning that changing this print orientation caused a decrease in the strength of the PLA specimens but caused an increase in the strength of the BMD-PLA specimens. One possible explanation is that the alignment of BMD-PLA along the path of extrusion promotes the compressive strength of the material. The mechanism by which the basalt powder supports load may be more effective tangent to the direction of material extrusion.

Due to the uniqueness of the tested composite, there is currently no relevant data on basalt moon dust—PLA composites with which to compare against. However, many models exist which attempt to predict the effective elastic modulus of composite materials. The simplest model, known as the Reuss model [36], describes the lower bound of the elastic modulus for a composite material under iso-stress conditions (applicable to particulates dispersed in matrix), given by Equation (3).

(3) $$E_{eff}=\frac{E_{m}E_{d}}{{fE}_{m}+{(1-f)E}_{d}}$$

where $E_{eff}$ is the effective elastic modulus of the composite, $E_{m}$ is the elastic modulus of the matrix, $E_{d}$ is the elastic modulus of the dispersion, and f is the volume fraction of the dispersion. Work by Luo provides more complex models, which account for elasticity relations and the Poisson effect [37]. For the iso-stress condition:

(4) $$E_{eff}=\frac{E_{m}E_{d}\left[fE_{m}\left(1-\nu_{d}\right)+E_{d}\left(1-f\right)\left(1-\nu_{m}\right)\right]}{E_{m}E_{d}\left[{\nu_{m}\left(1-f\right)}^{2}\left(1-\nu_{d}\right)+{\nu_{d}f}^{2}\left(1-\nu_{m}\right)\right]+\;f\left(1-f\right){E_{m}}^{2}\left[\left(1+\nu_{d}\right)\left(1-2\nu_{d}\right)\right]+4\nu_{d}\nu_{m}E_{d}E_{m}+(1+\nu_{m})(1-2\nu_{m})E_{d}^{2}\;}$$

where $\nu_{m}$ is Poisson’s ratio of the matrix material and $\nu_{d}$ is Poisson’s ratio for the dispersed particles. These two models were used in conjunction with area fraction data and material properties for the constituent materials to compare calculated elastic modulus values with the observed values. The volume fraction was taken from the average of the ImageJ analysis results, 30.9%. Poisson ratio values for PLA and lunar basalt simulants are well defined, as are elastic modulus values for PLA. For PLA, the elastic modulus was taken to be 2.54 GPa [35]. Poisson ratios were taken to be 0.36 and 0.246 for PLA and the basalt simulant, respectively [38,39]. However, there is a very wide range of data on elastic modulus values for basalt simulants, mostly dependent on the porosity of the samples tested. Each of the models were compared using modulus values from multiple literature sources, and the results are shown in Table 8.

Luo’s improved Reuss model predicts the closest values to our observed effective elastic moduli, but all models fall outside the range seen in Table 4. Since our observed PLA elastic modulus values are similar to the accepted values from the literature (2.45–2.61 GPa), it is unlikely that the modulus differences are due to the 3D printing geometry. Across all print parameters except batch 2, BMD-PLA displayed higher elastic modulus values than PLA. The highly jagged nature of the particulates, shown in Figure 5, may play a role in reducing the effective modulus beyond what current models predict. Additionally, if particle–matrix interface adhesion is particularly poor compared with typical composites, this could lead to reduced load transfer, resulting in lower-than-expected elastic modulus values.

In our analysis, it must be noted that conventional mechanical testing standards do not consider some of the complexities of 3D-printed parts. The ASTM D695 [35] is designed for isotropic, continuous cross-section polymer specimens. Due to infill geometry, some of the specimens have non-prismatic cross-sections, and it may be worth considering the varying cross-sectional area as a possible factor in the deformation behavior.

**Figure 7:**
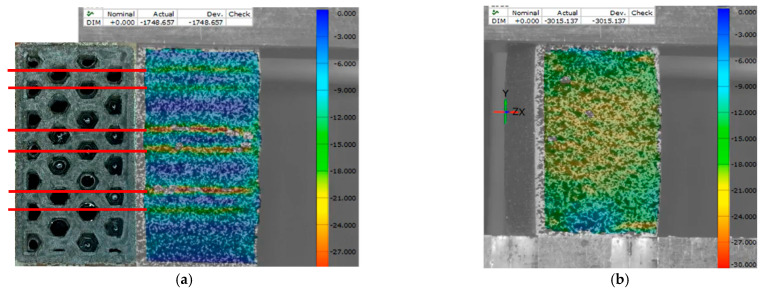
DIC strain gauge images. (**a**) Cross-sectional cut superimposed on DIC strain map of batch 2; (**b**) batch 6 strain map near ultimate load.

In Figure 7a, an image of the specimen cross-section is superimposed on the side of a still image of the strain map at just before maximum load. It shows the internal structure of the specimen which has 50% infill and 90° orientation, with red lines which extend the high strain regions. The lines of high strain fall along regions where the cross-section appears to contain more voids due to the honeycomb infill structure dictated.

In Figure 7b, a 100% infill sample is shown, which did not have concentrated regions of high strain, but rather a gradient across the surface. The 50% infill, 0° sample shows similar behavior to the 100% samples but with higher peak strain. These large strain concentrations could explain the much lower values of strain at ultimate load for BMD-PLA in 90° (see Appendix A).

**Figure 8:**
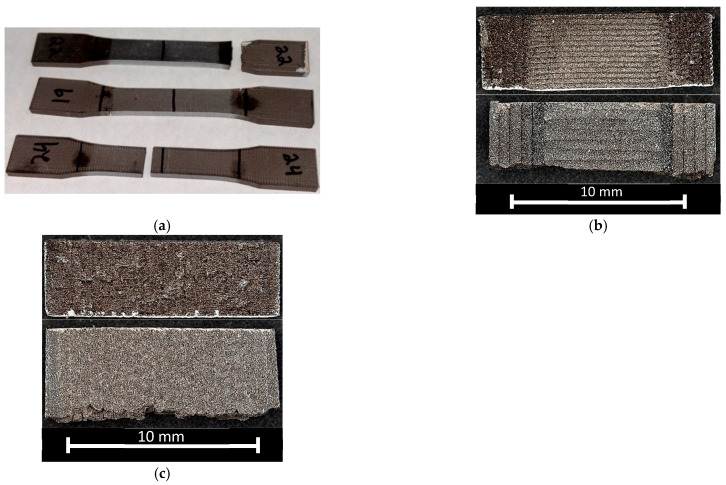
(**a**) Tensile samples with varying failure modes. The top sample (90° infill) had a clean break at the top grip. The middle sample (90° infill) had two incomplete shears on opposite sides. The bottom sample (0° infill) had a clean break within the gauge length; (**b**) batch 1 fracture surface and profile; (**c**) batch 9 fracture surface and profile.

Tensile tests were conducted for both BMD-PLA and PLA, but difficulties arose in obtaining consistent breakage/deformation within the gauge length of the BMD-PLA specimens. The material behaves with much less ductility in this test condition compared with compression, causing many samples to break prematurely or at/near the test stand grips. This is likely due to unintended bending moments being applied by small misalignment within the grips, which is a well-known difficulty with tensile testing of ceramics [42]. Figure 8 shows the various failure points of a selection of tensile tests. The 0° tensile samples would break outside the gauge length more frequently than the 90° samples and had much smoother fracture surfaces due to delamination at the layer lines.

**Figure 9:**
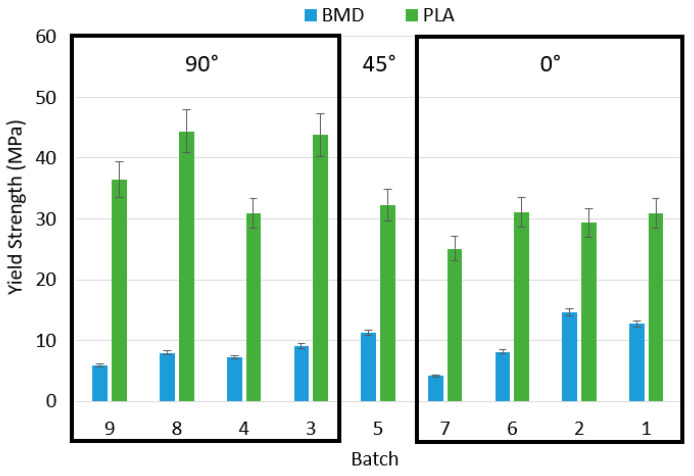
Comparison of tensile yield strength for BMD-PLA infill.

For this reason, the quality of the tensile response values for elastic modulus, yield, and ultimate strength are called into question. However, the discussion will still compare a limited set of acceptable BMD-PLA tensile samples with their PLA counterparts qualitatively. Comparing the overall behavior of the PLA and BMD-PLA tensile data corroborates the relationship between pure matrix and composite that was noted in compression. A graph of PLA and BMD tensile yield strengths is given in Figure 9, organized from 0° infill to 90° infill.

Batches 3, 4, 8, and 9 all have 90° infill. Batch 5 has a 45° alternating infill. Batches 1, 2, 6, and 7 have 0° infill. This orientation change again resulted in an overall decrease in PLA strength, but an increase in BMD-PLA strength. This could suggest that the particulates are able to improve the interface strength between individual strands of extruded filament within the infill. Note that the largest and smallest yield strength for BMD-PLA were both specimens with one shell, suggesting that shell count does not have a linear influence on tensile strength. Further work is needed to verify this, perhaps by utilizing a ceramic-specific tensile test standard rather than one for polymers.

Batch 2 was the strongest BMD-PLA configuration in tension. This corresponds to 0-degree infill, one shell, and 0.3 mm layer height. One possible explanation for this is that the composite material performs best with most of its internal geometry aligned in the axis of loading. Batch 1 saw similar strength values with five shells, which are all primarily aligned in the loading axis, meaning the gauge lengths of batch 1 and 2 are effectively identical.

## 4. Conclusions

Our future presence on the moon depends upon the ability to utilize the resources available on the lunar surface. To this end, the lunar regolith has been shown to have a variety of uses for construction and radiation shielding. Additionally, additive manufacturing methods which have become widespread in the space industry may be capable of utilizing in situ materials. This work explored a processing method using FFF additive manufacturing of a basalt moon dust-PLA simulant composite material. The composite filament possessed lower yield and ultimate strength properties than pure PLA in both compression and tension. The observed elastic modulus of the BMD-PLA is lower than that predicted by the rule of mixtures. If these materials are to be used for in situ manufacturing, processes will need to be adjusted to account for this. Additionally, qualitative analysis shows that the particulates do more than just “take up space” in the matrix material; BMD-PLA’s strength appears to benefit from certain 3D printing parameters that would normally be detrimental to pure PLA, specifically layer orientation. This may be important for informing design choices when these materials are utilized in situ. Further work is needed to validate alternate tensile testing methods that are designed for ceramics.

## Figures and Tables

**Table 1:** Basalt moon dust filament composition (data provided by The Virtual Foundry).

Material	% wt.
Basalt	46.2–47.74
Ilmenite	8.64–8.93
Anorthosite	5.16–5.33
Binding Additive	-
PLA	<40.00

**Table 2:** Compression testing details of experimental batches with a 0.2 mm print layer height.

Batch	Infill %	Shells	Orientation
1	50	1	0
2	50	1	90
3	50	3	0
4	50	3	90
5	100	1	0
6	100	1	90
7	100	3	0
8	100	3	90

**Table 3:** Tensile testing details of experimental batches with 100% infill.

Batch	Infill Angle	Shells	Layer Height
1	0	5	0.3
2	0	1	0.3
3	90	5	0.3
4	90	1	0.3
5	45	3	0.2
6	0	5	0.1
7	0	1	0.1
8	90	5	0.1
9	90	1	0.1

**Table 4:** PLA compression response values.

Batch	σ_y_ [MPa]	σ_ult_ [MPa]	E [GPa]
1	23.2 ± 0.6	-	1.15 ± 0.05
2	21.0 ± 1.2	-	1.03 ± 0.04
3	37.5 ± 1.0	-	1.72 ± 0.04
4	29.1 ± 0.5	-	1.45 ± 0.03
5	62.2 ± 2.6	-	2.61 ± 0.12
6	47.6 ± 0.9	-	2.45 ± 0.06
7	63.1 ± 1.6	-	2.58 ± 0.07
8	49.3 ± 0.3	-	2.48 ± 0.04

**Table 5:** BMD-PLA compression response values.

Batch	σ_y_ [MPa]	σ_ult_ [MPa]	E [GPa]
1	8.82 ± 0.2	23.1 ± 0.3	0.93 ± 0.08
2	10.6 ± 0.6	13.6 ± 0.2	1.15 ± 0.10
3	8.17 ± 0.2	28.0 ± 0.3	0.91 ± 0.08
4	10.1 ± 0.5	13.6 ± 0.9	1.27 ± 0.10
5	23.4 ± 1.0	54.9 ± 0.3	2.41 ± 0.18
6	27.7 ± 0.4	41.8 ± 0.8	2.04 ± 0.12
7	18.6 ± 0.3	47.4 ± 0.4	2.16 ± 0.26
8	21.0 ± 1.0	39.8 ± 1.2	2.22 ± 0.23

**Table 6:** Area fraction values. Images are included in Appendix C.

Image Filename	Area %
BMD8-0 150-0001.jpg	33.418
BMD5-0 150-0001.jpg	33.929
BMD5-90 150-0001.jpg	34.381
BMD6-90 150-0001.jpg	24.224
BMD4-0 500-Shell0001.jpg	33.414
BMD7-0 150-0001.jpg	26.108

**Table 7:** The 50% infill specimen surface area fraction values.

Batch (Parameters)	Solid Surface Area %
Batch 1 (0°, 1 shell)	74.8
Batch 2 (90°, 1 shell)	66.1
Batch 3 (0°, 3 shell)	85.4
Batch 4 (90°, 3 shell)	80.1

**Table 8:** Predicted effective modulus of BMD-PLA.

PLA Elastic Modulus [GPa]		2.54 [35]		
Lunar Simulant Elastic Modulus [GPa]		108.9 [40]	46.2 [41]	8.4 [39]
BMD-PLA Effective Modulus [GPa]	Reuss Model	7.81	7.32	4.89
	Luo Model	5.51	5.10	3.65

**Figure A1:**
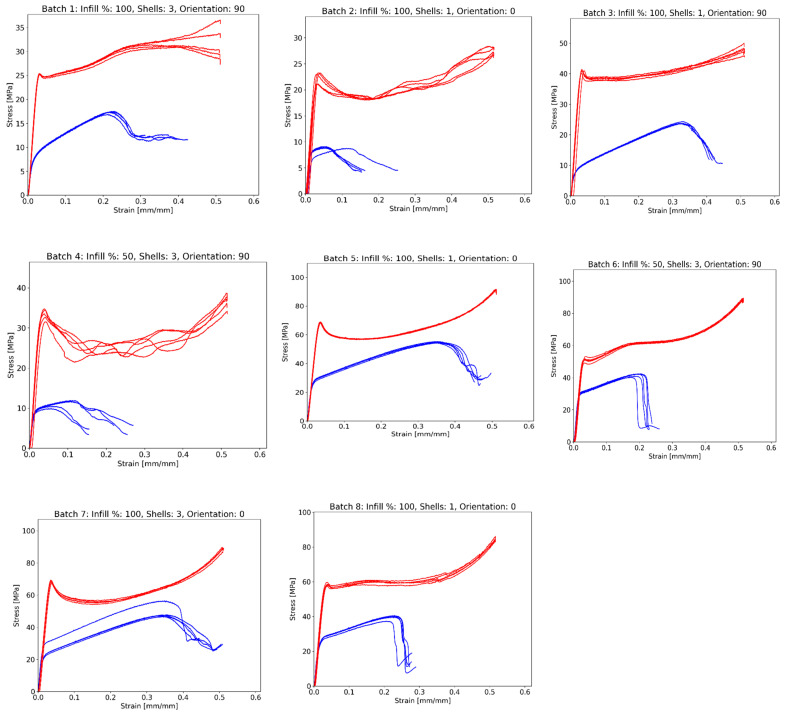
Collated stress–strain curves for each batch of compression tests. Five samples were tested from each batch. Red—PLA; blue—BMD-PLA.

**Figure A2:**
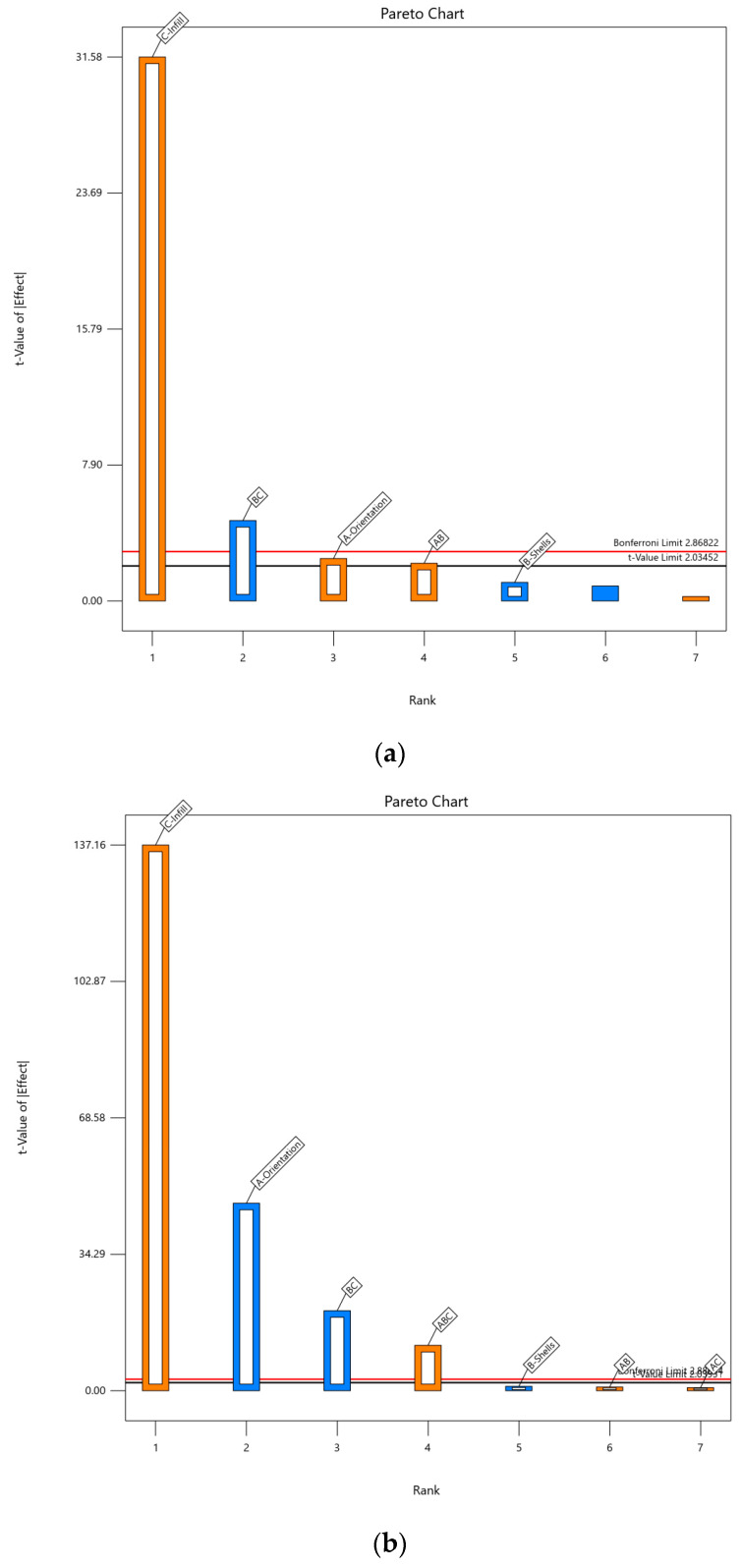

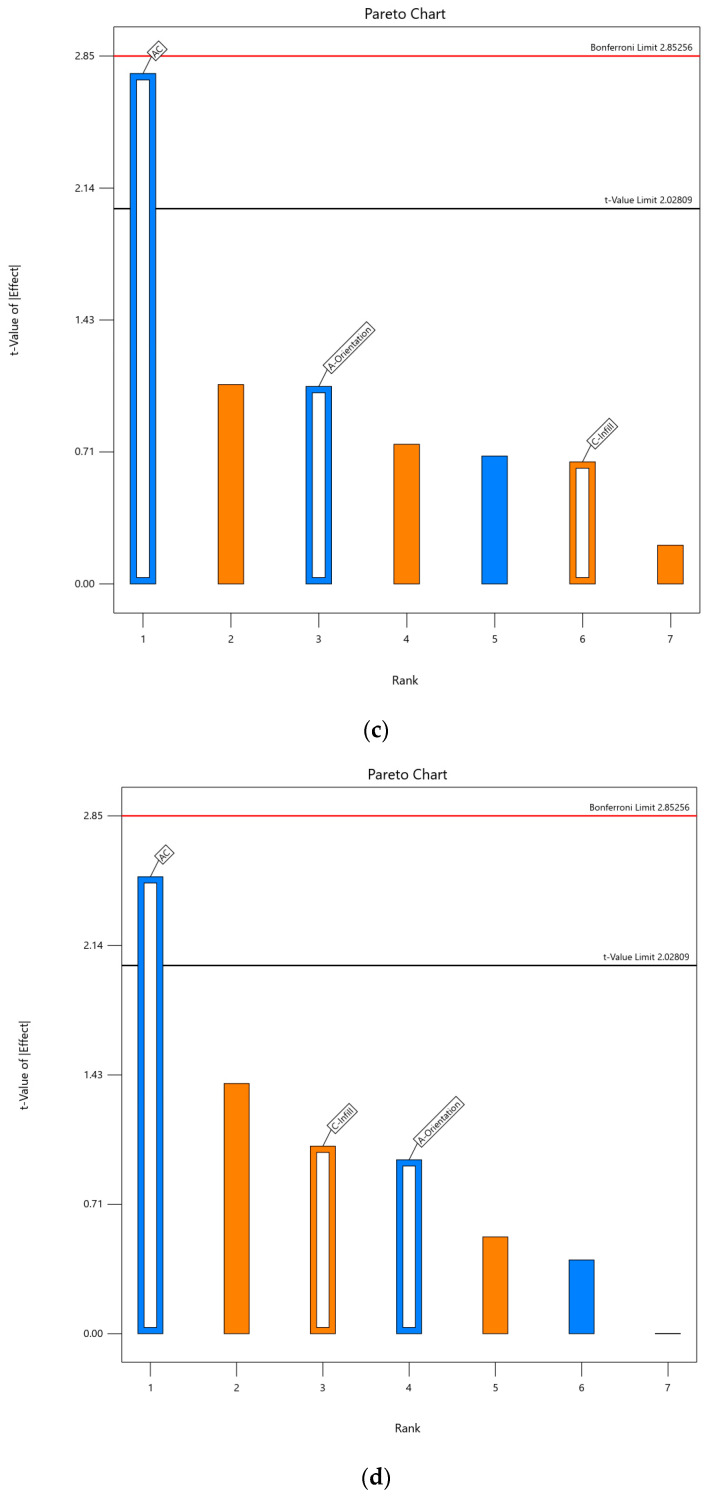
(**a**) BMD-PLA elastic modulus; (**b**) BMD-PLA ultimate strength; (**c**) PLA yield strength; (**d**) PLA elastic modulus.

**Figure A3:**
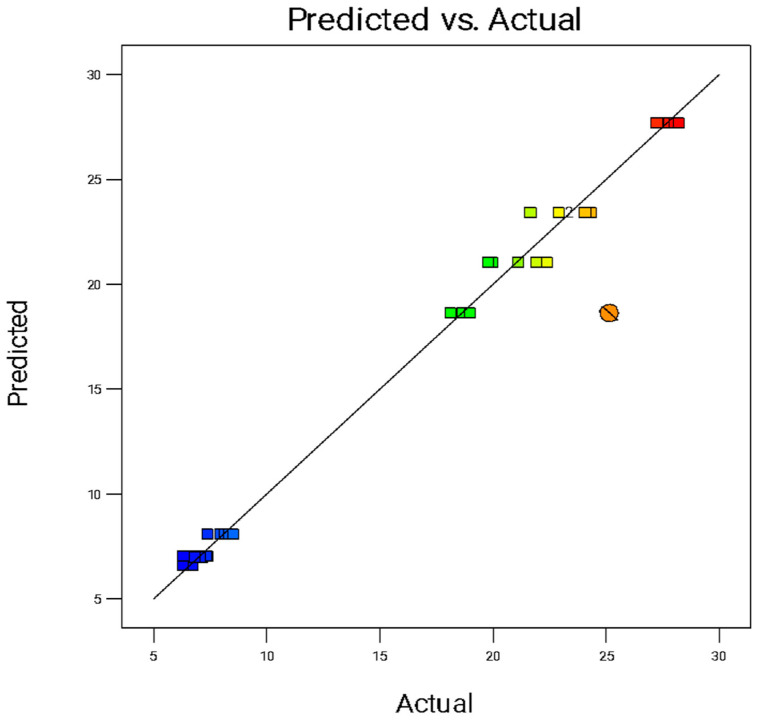
Fitted vs. actual plot for model of BMD-PLA yield strength. The color gradient indicates a heatmap from lowest (blue) to highest (red) experimental value.

**Figure A4:**
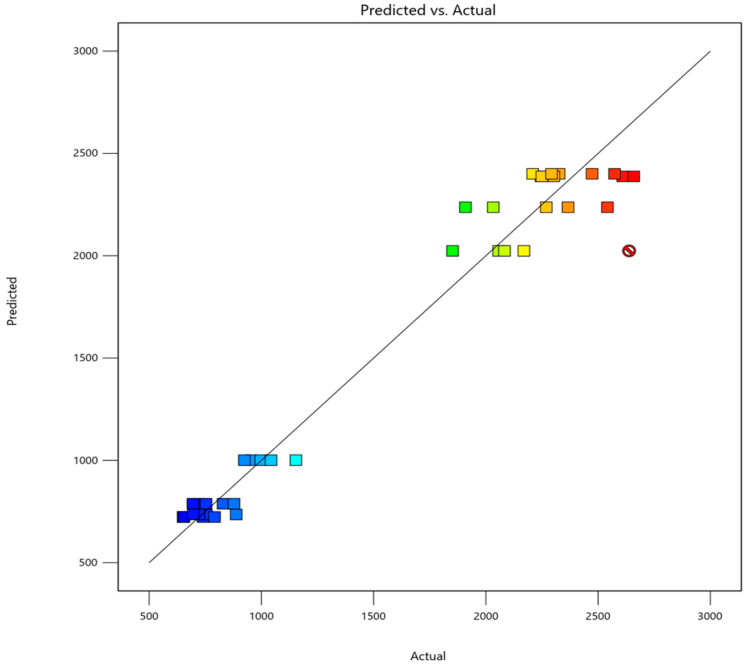
Fitted vs. actual plot for model of BMD-PLA elastic modulus.

**Figure A5:**
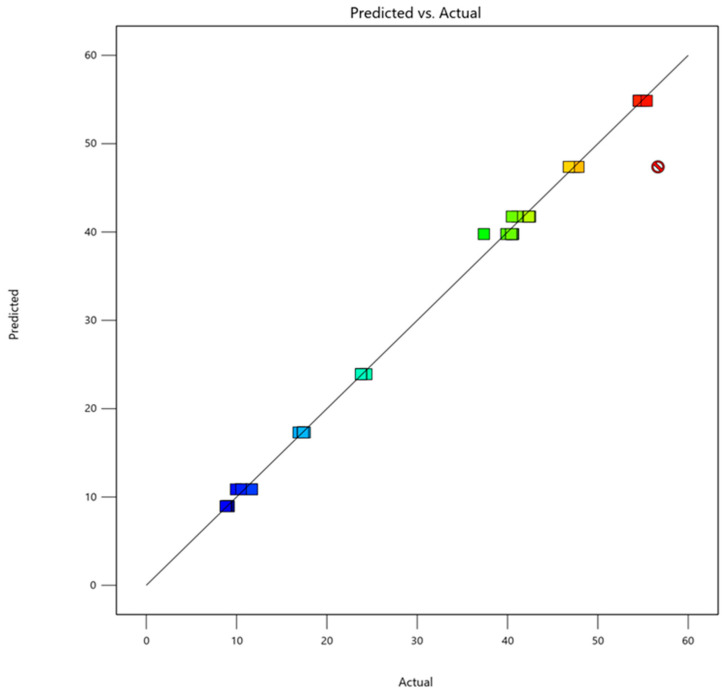
Fitted vs. actual plot for model of BMD-PLA ultimate strength.

## Data Availability

Data are contained within the article.

## Abbreviations

The following abbreviations are used in this manuscript:

ASTM	American Society for Testing and Materials
BMD	Basalt Moon Dust
DoE	Design of Experiments
ISRU	In Situ Resource Utilization
PLA	Polylactic Acid
SEM	Scanning Electron Microscope

## Appendix B

### Appendix B.1

**Table A1:** *p*-values of factors and interactions for BMD-PLA compression tests.

	Yield Strength	Ultimate Strength	Young’s Modulus
Model	<0.0001	<0.0001	<0.0001
A—Orientation	<0.0001	<0.0001	0.0194
B—Shells	<0.0001	0.2917	0.2897
C—Infill	<0.0001	<0.0001	<0.0001
AxB	0.1658	0.3707	0.0361
AxC	<0.0001	0.4571	-
BxC	<0.0001	<0.0001	<0.0001
AxBxC	0.005	<0.0001	-

### Appendix B.2

**Table A2:** *p*-values of factors and interactions for PLA compression tests.

	Yield Strength	Ultimate Strength	Young’s Modulus
Model	0.0405	N/A	0.0554
A—Orientation	0.2930	N/A	0.3442
B—Shells	-	N/A	-
C—Infill	0.5140	N/A	0.3084
AxB	-	N/A	-
AxC	0.0091	N/A	0.0164
BxC	-	N/A	-
AxBxC	-	N/A	-
